# Supplementary material for: Effects of Probiotic Supplementation on Exercise with Predominance of Aerobic Metabolism in Trained Population: A Systematic Review, Meta-Analysis and Meta-Regression
Source: Nutrients. 2022 Jan 30;14(3):622. doi: 10.3390/nu14030622 (PMC8840281; doi:10.3390/nu14030622)
Supplement: Supplementary file 1 [file nutrients-14-00622-s001.zip › Supplementary file 1.pdf]

Table S1: Code lines for search in each database

PubMed/MEDLINE database:<br>("probiotics"[MeSH Terms] OR "probiotics"[All Fields]) AND (("exercise"[MeSH Terms] OR "exercise"[All Fields]) OR ("sports"[MeSH Terms] OR "sports"[All Fields] OR "sport"[All Fields]) OR performance[All Fields] OR aerobic[All Fields]) AND (("athletes"[MeSH Terms] OR "athletes"[All Fields] OR "athlete"[All Fields]) OR trained[All Fields] OR elite[All Fields]).
WOS database:<br>(Probiotics and (exercise or sport or performance or aerobic) and (athlete or trained or elite)).
Scopus database:<br>(Probiotics and (exercise or sport or performance or aerobic) and (athlete or trained or elite)).
