# Supplementary material for: Effects of Probiotic Supplementation on Exercise with Predominance of Aerobic Metabolism in Trained Population: A Systematic Review, Meta-Analysis and Meta-Regression
Source: Nutrients. 2022 Jan 30;14(3):622. doi: 10.3390/nu14030622 (PMC8840281; doi:10.3390/nu14030622)
Supplement: Supplementary file 1 [file nutrients-14-00622-s001.zip › Supplementary file 2.pdf]

Table S2: The reason of excluded articles

Article	Reason for exclusion
Effects of Lactobacillus plantarum TWK10 on Exercise Physiological Adaptation, Performance, and Body Composition in Healthy Humans	Not highly trained population
The effects of postexercise consumption of a kefir beverage on performance and recovery during intensive endurance training	Not highly trained population
Bifidobacterium animalis subsp. lactis BB-12 Improves the State Anxiety and Sports Performance of Young Divers Under Stress Situations: A Single-Arm, Prospective Proof-of-Concept Study.	Not highly trained population
Probiotic Bacillus coagulans GBI-30, 6086 reduces exercise-induced muscle damage and increases recovery	Not highly trained population
The effects of combined probiotic ingestion and circuit training on muscular strength and power and cytokine responses in young males	Not highly trained population
The Effects of Plasmacytoid Dendritic Cell-Stimulative Lactic Acid Bacteria, Lactococcus lactis Strain Plasma, on Exercise-Induced Fatigue and Recovery via Immunomodulatory Action	Not performance measure
Effect of a probiotic intake on oxidant and antioxidant parameters in plasma of athletes during intense exercise training	Not performance measure
Probiotic supplementation elicits favourable changes in muscle soreness and sleep quality in rugby players.	Not performance measure
Effects of Daily Probiotics Supplementation on Anxiety Induced Physiological Parameters among Competitive Football Players	Not performance measure
The Bifidobacterium bifidum (BIB2) Probiotic Increased Immune System Factors in Men Sprint Athletes	Not performance measure
Efficacy of heat-killed Lactococcus lactis JCM 5805 on immunity and fatigue during consecutive high intensity exercise in male athletes: a randomized, placebo-controlled, double-blinded trial	Not performance measure
The Effects of Plasmacytoid Dendritic Cell-Stimulative Lactic Acid Bacteria, Lactococcus	Not performance measure

lactis Strain Plasma, on Exercise-Induced Fatigue and Recovery via Immunomodulatory Action.	
Effect of Multi-Strain Probiotic Supplementation on URTI Symptoms and Cytokine Production by Monocytes after a Marathon Race: A Randomized, Double-Blind, Placebo Study.	Not performance measure
Effects of Probiotic (Bacillus subtilis DE111) Supplementation on Immune Function, Hormonal Status, and Physical Performance in Division I Baseball Players	No aerobic performance measure
The Effect of 2 Weeks of Inactivated Probiotic Bacillus coagulans on Endocrine, Inflammatory, and Performance Responses During Self-Defense Training in Soldiers	No aerobic performance measure
Effects of Probiotic (Bacillus subtilis) Supplementation During Offseason Resistance Training in Female Division I Athletes	No aerobic performance measure
Probiotic Streptococcus thermophilus FP4 and Bifidobacterium breve BR03 Supplementation Attenuates Performance and Range-of-Motion Decrements Following Muscle Damaging Exercise	No aerobic performance measure
